# Individual Radiosensitivity in Oncological Patients: Linking Adverse Normal Tissue Reactions and Genetic Features

**DOI:** 10.3389/fonc.2019.00987

**Published:** 2019-10-01

**Authors:** Elisa Palumbo, Celeste Piotto, Enrica Calura, Elena Fasanaro, Elena Groff, Fabio Busato, Badr El Khouzai, Michele Rigo, Laura Baggio, Chiara Romualdi, Demetre Zafiropoulos, Antonella Russo, Maddalena Mognato, Luigi Corti

**Affiliations:** ^1^Department of Radiotherapy, Veneto Institute of Oncology IOV–IRCCS, Padua, Italy; ^2^Department of Biology, University of Padua, Padua, Italy; ^3^National Laboratories of Legnaro, Italian Institute of Nuclear Physics (LNL-INFN), Padua, Italy; ^4^Department of Molecular Medicine, University of Padua, Padua, Italy

**Keywords:** radiotherapy, adverse effects, chromosomal radiosensitivity, gene expression, association analysis

## Abstract

**Introduction:** Adverse effects of radiotherapy (RT) significantly affect patient's quality of life (QOL). The possibility to identify patient-related factors that are associated with individual radiosensitivity would optimize adjuvant RT treatment, limiting the severity of normal tissue reactions, and improving patient's QOL. In this study, we analyzed the relationships between genetic features and toxicity grading manifested by RT patients looking for possible biomarkers of individual radiosensitivity.

**Methods:** Early radiation toxicity was evaluated on 143 oncological patients according to the Common Terminology Criteria for Adverse Events (CTCAE). An individual radiosensitivity (IRS) index defining four classes of radiosensitivity (highly radiosensitive, radiosensitive, normal, and radioresistant) was determined by a G_2_-chromosomal assay on *ex vivo* irradiated, patient-derived blood samples. The expression level of 15 radioresponsive genes has been measured by quantitative real-time PCR at 24 h after the first RT fraction, in blood samples of a subset of 57 patients, representing the four IRS classes.

**Results:** By applying univariate and multivariate statistical analyses, we found that fatigue was significantly associated with IRS index. Interestingly, associations were detected between clinical radiation toxicity and gene expression (*ATM, CDKN1A, FDXR, SESN1, XPC, ZMAT3*, and *BCL2/BAX* ratio) and between IRS index and gene expression (*BBC3, FDXR, GADD45A*, and *BCL2/BAX*).

**Conclusions:** In this prospective cohort study we found that associations exist between normal tissue reactions and genetic features in RT-treated patients. Overall, our findings can contribute to the identification of biological markers to predict RT toxicity in normal tissues.

## Introduction

The development of radiation-induced complications following radiotherapy (RT) has a significant impact on treatment outcome and patient's quality of life (QOL). In the last decades the therapeutic ratio has improved due to advancements in RT technologies and use of radioprotectors, mitigators, and radiosensitizers (1). Nevertheless, radiation toxicity of normal tissues surrounding the tumor is a serious problem for ~5–10% of patients, who are affected by high intrinsic radiosensitivity (2–4). Evidence of radiosensitivity *in vivo* is given by burns and radiodermitis in the irradiated body parts, together with bystander effect in neighboring area (5). Several factors, including cellular composition, differentiation, cell renewal capacity, as well as cellular radiosensitivity, determine the severity of radiation toxicity (6). Patient-related factors are deeply linked to the risk of manifesting radiation toxicity, and reliable biological markers are still not available to predict the onset of severe side-effects after RT. Human response to ionizing radiation (IR) is individual and variable, being influenced by age, smoking, diabetes, collagen vascular disease and genotype (7). Moreover, multiple genetic pathways such as DNA damage repair, oxidative stress, radiation fibrogenesis and endothelial cell damage are implicated in adverse tissue reactions following radiotherapy (8). However, the molecular basis of individual radiosensitivity remains poorly understood, and the relationship between different indicators of radiation sensitivity is elusive.

RT causes cancer cell death mainly by IR-induced DNA Double Strand Breaks (DSBs). Formation of DSBs, the most severe damage for genome integrity, triggers a cascade of cellular events, collectively termed DNA-damage response (DDR), which involves sensing the damage, signal transduction to the effectors of DNA repair, cell cycle arrest and apoptosis induction (9–11). Radiation-induced DSBs are efficiently repaired to ensure the maintenance of genome integrity but when DNA repair is hampered, unrepaired DSBs can originate chromosome aberrations (12). Following irradiation, unrepaired DSBs can be quantified in metaphase spreads by the yield of chromatid breaks formed at G_2_-phase, which is inversely related to the efficiency of the G_2_-phase checkpoint (13). Thus, the individual level of radiosensitivity can be assessed in *ex vivo* irradiated human peripheral blood lymphocytes (PBLs) by applying a “G_2_-chromosomal assay” (14–17).

Increasing evidence supports the existence of individual response to IR-induced DNA damage, which can be related to mutations in key genes of DDR pathway or to individual capacity to modulate the expression of DDR genes after IR-exposure. In this regard, the expression of genes involved in DDR pathway may be variable between individuals and can impact on own radiation response.

Several studies attempted to find biomarkers able to predict the onset of radiation toxicity in normal tissues after RT. Individual radiosensitivity evaluated by using *in vitro* irradiated patient-derived blood lymphocytes has been found to correlate with normal tissue reactions (13, 18, 19), and single nucleotide polymorphisms (SNPs) have been associated with acute and late radiation-induced normal tissue injury in RT patients (19–22). Data concerning the association between gene expression changes and normal tissue radiation toxicity refer to *in vitro* irradiation studies (4, 23–25) or to single gene analysis in oncological patients treated with RT (26). To date, a relationship between radio-induced normal tissue adverse effects, *in vitro* chromosomal radiosensitivity and *in vivo* expression of a set of radioresponsive genes is not available in the same cohort of RT patients. Since future clinical protocols aim at ameliorating patient's QOL it is demanding to identify patient-related factors that are associated with individual radiosensitivity before patients undergo RT (27, 28).

In this explorative study, the clinical features of early radiation toxicity have been associated with an Individual Radiosensitivity (IRS) index, defining four classes of radiosensitivity (highly radiosensitive, radiosensitive, normal and radioresistant) based on a G_2_-chromosomal assay on patient-derived PBLs irradiated *in vitro* (15). The expression level of 15 selected radioresponsive genes belonging to DDR pathway has been measured in blood samples from a subgroup of patients, representing the different IRS classes, 24 h after the first RT fraction, as an additional variable of intrinsic radiosensitivity. Data of clinical and genetic features have been statistically analyzed to find possible genetic factors associated with individual radiation sensitivity.

## Materials and Methods

### Outline of the Study

**Figure 1:**
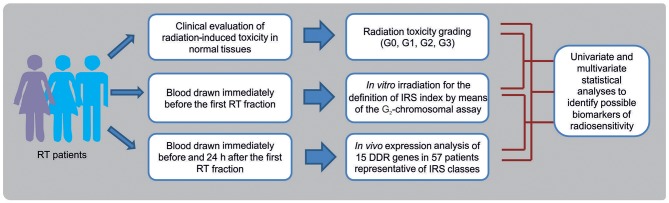
Flow chart showing the experimental phases for the identification of biomarkers of individual radiosensitivity in patients undergoing radiotherapy (RT).

In this prospective study, breast cancer (BC) and head and neck squamous cell carcinoma (HNSCC) patients were enrolled as representative of patients experiencing normal tissue reactions after RT. Data of toxicity grading in normal tissues, *in vitro* chromosomal radiosensitivity and *in vivo* RT-induced gene expression changes, have been integrated to identify possible biomarkers of radiosensitivity in patients undergoing RT (Figure 1). Overall, 143 oncological patients were enrolled: 124 (all females) affected by BC, and 19 (6 females and 13 males) affected by HNSCC.

### Patients

Patients with BC or HNSCC histological diagnosis undergoing RT were enrolled from 2015 to 2017 at the Department of Radiotherapy, Veneto Institute of Oncology IOV–IRCCS, Padua, Italy (IOV) upon evaluation and approval of the IOV-IRCCS Ethic Committee (CE IOV 2015/18; CE IOV 2016/04). Privacy rights of human subjects were observed; all the procedures were in accordance with relevant guidelines and regulations. All subjects gave written informed consent in accordance with the Declaration of Helsinki.

Patients were enrolled applying the following exclusion criteria: patients suffering from congenital syndromes predisposing to radiosensitivity (such as Ataxia-Telangiectasia, Bloom syndrome, Down's syndrome, Gorlin syndrome, Klinefelter syndrome, Retinoblastoma, Wilm's tumor, Xeroderma pigmentosum, Rothmund-Thomson syndrome, Li-Fraumeni syndrome, Dyskeratosis congenita, Familial dysplastic nevus syndrome, Common variable immune deficiency, Nijmegen Breakage Syndrome, Fanconi Anemia, albinism), previous RT and/or chemotherapy treatment or ongoing chemotherapy treatment, previous anticancer drug employment, significant comorbidities, diabetic patients affected by breast cancer, age ≤ 18 years.

BC patients received by 3DCRT (42.40–50 Gy/16–25 fractions) plus a boost dose of 10 Gy in 5 fractions to the tumor bed. HNSCC patients received up to 70 Gy, daily fraction 1.8–2.12 Gy/day, for 5 days/week on primitive tumor by VMAT or IMRT.

Adverse tissue reactions (dermatitis radiation, pain, pruritus, fatigue) have been recorded at the completion of RT treatment (t_1_) and 1 month later (t_2_), using the Common Terminology Criteria for Adverse Events (CTCAE) (version 4.03, http://ctep.cancer.gov/protocolDevelopment/adverse_effects.htm). Adverse effects were classified as: grade 0 (G0, no adverse effects), grade 1 (G1, mild), grade 2 (G2, moderate), grade 3 (G3, severe). At the Department of Radiotherapy of the IOV-IRCCS management of acute toxicity followed a standardized procedure. All patients were clinically evaluated before starting RT and no significant side-effects were complained by patients.

### Chromosome-Based Radiosensitivity Assay

The G_2_-chromosomal assay was performed following a standardized protocol (15). Briefly, whole blood cultures were incubated for 72 h at 37°C, 5% CO_2_ before being irradiated with 1 Gy of gamma rays in a Gamma Beam A15 ^60^Co panoramic source at the National Laboratories of Legnaro (I.N.F.N., Padua, Italy; dose rate: 0.5 Gy/min).

Immediately after irradiation, each culture was split in two and one was treated with 4 mM caffeine. After 20 min at 37°C, both cultures were incubated with Colcemid at concentration of 0.1 μg/mL for 60 min, then chromosome spreads were prepared according to standard cytogenetic procedures. With few exceptions, chromatid aberration yields were obtained by scoring for chromatid breaks and gaps 50 metaphases per culture, under a Zeiss AxioImager Z2 microscope coupled with MSearch-AutoCapt software (Metasystems, Altlussheim Germany). Following calculation of the *in vitro* individual radiosensitivity index (IRS = [1-(G_2caf_-G_2_)/G_2caf_] × 100%, simplified as IRS = (G_2_/G_2caf_) × 100%) patients were classified as: highly radiosensitive, HRS (IRS > 70), radiosensitive, RS (50 < IRS ≤ 70), normal, N (30 ≤ IRS ≤ 50), and radioresistant, RR (IRS < 30) (15, 29).

### Gene Expression Analysis

Fifty-seven over 143 patients were randomly selected within the four IRS classes (HRS, RS, N, RR) in order to have comparable numbers of patients in each group. This sample size guarantees a high statistical power (power = 0.83) in identifying as significant (alpha < 0.1) genes with an effect equal to 1.1 among groups using either an ANOVA test or a Wilcoxon test. Two whole blood samples were collected from each patient: one immediately before the first fractionated RT dose and the second 24 h later. Samples were collected into PAXgene® Blood RNA tubes (PreAnalytiX GmbH, Qiagen, Venlo, The Netherlands) for immediate stabilization of intracellular RNA, and stored at −80°C. Total RNA was purified by using PAXgene® Blood RNA Kit 6 (PreAnalytiX GmbH, Qiagen, Venlo, The Netherlands) and quantified using the ND-1000 spectrophotometer (Nanodrop, Wilmington, DE, USA).

For mRNA detection, retrotranscription and quantitative real time-PCR (qRT-PCR) reactions were performed according to our established protocol (30, 31). The gene-specific primers for *ATM, BAX, BBC3, BCL2, CCNG1, cMYC, DDB2, FDXR, GADD45A, MDM2, CDKN1A, PCNA, SESN1, XPC*, and *ZMAT3* genes and for *GADPH* as reference, can be found in Supplementary Table 1. Real-time PCR was performed using an Applied Biosystems 7500 Fast Real-Time PCR System according to the following amplification protocol: 95°C for 10 min, 95°C for 15 sec, 60°C for 60 s (40 cycles). qRT-PCR reactions were always performed in triplicates. The relative expression levels of mRNAs between irradiated (2 Gy) and non-irradiated (0 Gy) blood samples of the same patients were calculated using the comparative delta CT (threshold cycle number) method (2^−ΔΔCT^) implemented in the 7500 Real Time System software (32).

### Statistical Analysis

ANOVA and ANOVA *post-hoc* with Bonferroni correction was used to assess the gene expression mean differences among groups of patients defined using clinical and IRS annotations. In case of group size lower than 10 patients Kruskall-Wallis/Wilcoxon test was used. Multivariate regression analyses were used to test the association of clinical annotations (explanatory variables) with IRS value (dependent variable). Unsupervised cluster analysis of gene expression data was performed using hierarchical cluster analysis with Euclidean distance and complete linkage. All the analyses were performed using the R programming language (version 3.4), and the Bioconductor software suite (version 3.6).

## Results

### Radiation-Induced Toxicity in RT-Treated Patients

**Figure 2:**
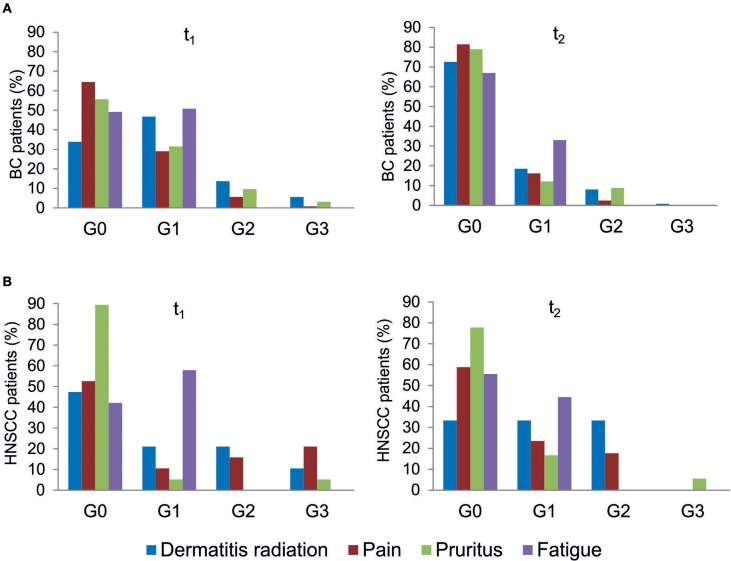
Clinical variables of radiation toxicity in RT patients. RT-induced toxicity grading in breast cancer, BC **(A)** and in head and neck squamous cell carcinoma HNSSC **(B)** patients at completion of RT treatment (t_1_) and 1 month later (t_2_).

All patients were evaluated for the onset of radiation toxicity at the completion of RT treatment (t_1_) and 1 month later (t_2_). The overall distribution of subjects suffering from dermatitis radiation, pain, pruritus and fatigue is reported in Figure 2; Supplementary Table 2. Moderate (G2) dermatitis radiation was recorded at t_1_ in 13.7 and 21% of BC and HNSCC patients, respectively, whereas severe (G3) dermatitis radiation was observed in 5.6 and 10.5% of BC and HNSCC, respectively. At t_2_, G2 dermatitis radiation was observed in 8% of BC patients and in 33% of HNSCC patients; G3 dermatitis radiation was manifested by 0.8% of BC patients. Pain of G2 grade was present in 5.6% of BC and in 15.8% of HNSCC patients at t_1_; 1 month later (t_2_), 2.4% of BC and 18% of HNSCC patients manifested G2 pain. At t_1_, G3 pain was rarely recorded in BC patients but affected 21% of the HNSCC patients; at t_2_ none of the BC and HNSCC patients suffered from pain of G3 grade. Pruritus of G2 and G3 grade was recorded at t_1_ in 9.7 and 3.2% of BC patients, respectively, while at t_2_, G2 pruritus was present in 8.9% of BC patients. Concerning HNSCC patients, 5.2 and 5.5% of them manifested G3 pruritus at t_1_ and t_2_, respectively. In summary, HNSCC patients manifested higher degrees of dermatitis radiation and pain at both t_1_ and t_2_, whereas pruritus appeared to be more pronounced in BC patients. Fatigue (G1 grade) was present in 50.8% (BC) and 57.8% (HNSCC) of patients, and at t_2_ in 33.0 and 44.5%, respectively (Figure 2).

### G_2_-Chromosomal Radiosensitivity in RT Patients

**Figure 3:**
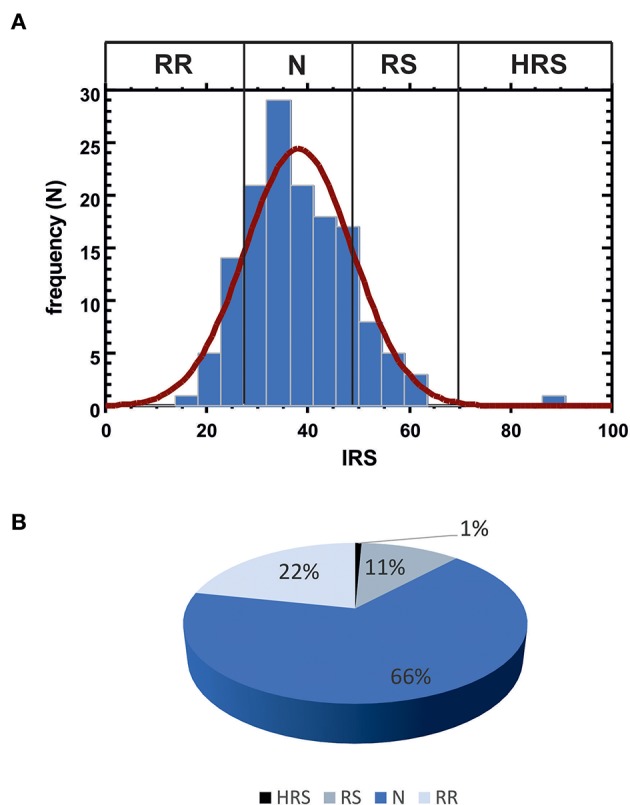
**(A)** Data distribution for IRS values. Vertical lines indicate the observed thresholds for the 4 classes of individual radiosensitivity, calculated as: RR, mean - SD (IRS < 27.48); N, mean ± SD (27.48 ≤ IRS ≤ 48.72); RS, mean + SD (48.72 < IRS ≤ 69.96); HRS, mean + 3 SD (IRS > 69.96). **(B)** Patient distribution according to the four IRS classes.

IRS values were determined in the complete patient cohort (143 subjects). In blood cultures exposed *in vitro* to 1 Gy [according to the standardized protocol developed by Pantelias and coworkers (15, 29)], the average yield of G_2_ chromatid breaks was 2.5 (with standard deviation SD = 0.084 and coefficient of variation CV = 3%); the average IRS was 38.1 (SD = 10.62; CV = 27.8%). Based on the observed distribution of individual IRS values, the four classes of individual radiosensitivity should be, respectively: RR < 27.48 (mean – SD); 27.48 ≤ N ≤ 48.72 (mean ± SD); 48.72 < RS ≤ 69.96 (mean + SD); HRS > 69.96 (mean + 3 × SD) (Figure 3A). As these values are in strict agreement with those proposed earlier (15), for further statistical analyses we used the published thresholds (see Materials and Methods). One patient resulted highly radiosensitive (HRS, 1%), 16 patients were classified as radiosensitive (RS, 11%), 95 patients as normal (N, 66%) and 31 as radioresistant (RR, 22%) (Figure 3B).

### Gene Expression in Blood Samples of RT Patients

Fifteen radioresponsive genes belonging to DDR pathway (Table 1) were analyzed by qRT-PCR in blood samples from 57 patients, randomly selected from the whole cohort, in order to have comparable numbers of patients within the four IRS classes (HRS, RS, N, and RR). A summary of the clinical data of this group of patients is available in Supplementary Table 3.

**Table 1:** Names and function of DDR genes evaluated by qRT-PCR in blood samples from RT patients.

**Gene symbol**	**Gene name**	**Function**
*ATM*	Ataxia Telangiectasia Mutated	DNA damage signal transduction; cell cycle checkpoint
*BAX*	BCL2-associated X protein	Apoptosis
*BBC3*	BCL2-binding component 3 (PUMA)	Apoptosis
*BCL2*	B-Cell CLL/Lymphoma 2	Apoptosis
*CCNG1*	Cyclin G1	Cell cycle progression/arrest
*CDKN1A*	Cyclin-dependent kinase inhibitor 1A (p21)	Cell cycle arrest
*cMYC*	MYC proto-oncogene, bHLH transcription factor	Cell cycle progression, apoptosis and cellular transformation
*DDB2*	Damage-specific DNA binding protein 2 (p48)	DNA repair
*FDXR*	Ferrodoxin reductase	DNA damage, apoptosis
*GADD45A*	Growth arrest and DNA-damage-inducible, alpha	Growth arrest; DNA repair; apoptosis
*MDM2*	Mdm2 p53 binding protein homolog	Inactivation of tumor protein p53
*PCNA*	Proliferating cell nuclear antigen	DNA repair
*SESN1*	Sestrin 1 (Sestrins)	Cell cycle arrest
*XPC*	Xeroderma pigmentosum, complementation group C	DNA repair
*ZMAT3*	Zinc finger, matrin type 3 (PAG608)	Cell growth; apoptosis

**Figure 4:**
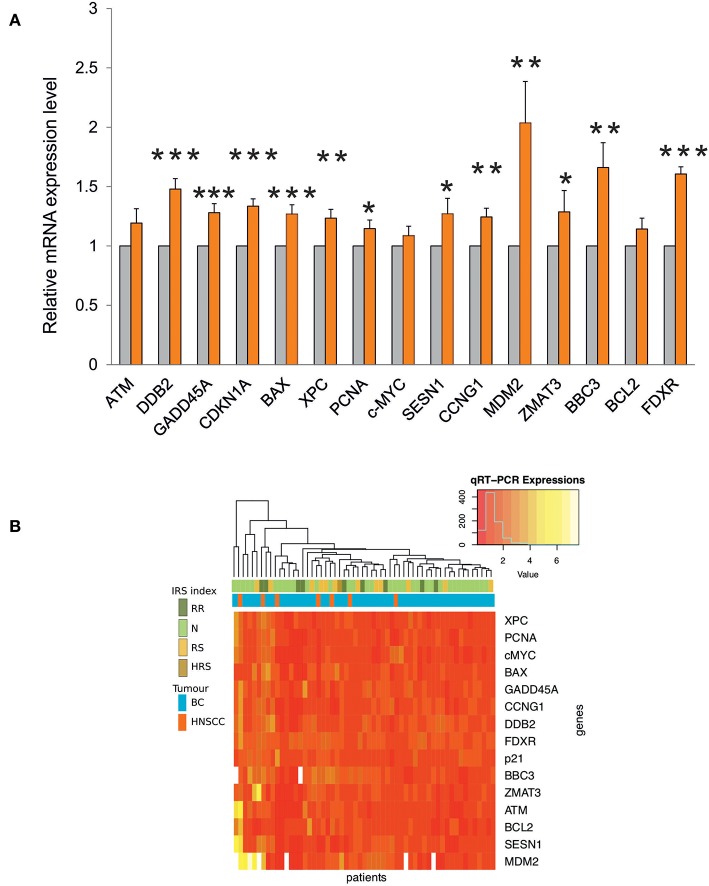
**(A)** Gene expression analysis by qRT-PCR in blood samples from RT patients. The relative mRNA quantification was performed by comparing irradiated vs. non-irradiated blood samples derived from the same patient. Values are mean ± SE and expressed in fold-change. The value “1” of non-irradiated control (light gray bars) is arbitrarily given when no change is observed (***p < 0.001; **p < 0.01; *p < 0.05). **(B)** Heatmap and unsupervised cluster analysis on the expression profiles of DDR genes analyzed in 57 RT patients. The key color bar indicates standardized gene expression levels (low levels are in red, high levels are in yellow). The annotation bars (upper part of the heatmap) indicate the four classes of IRS index and tumor types.

Transcription of most genes was significantly induced after the first RT fraction (Figure 4A).

The unsupervised cluster analysis of gene expression profiles reported as a heatmap in Figure 4B did not reveal differences between the two types of cancer, although the response of DDR genes was variable across patients. Indeed, a group of patients is characterized by high expression values of *MDM2, SESN1, BCL2, ATM*, and *ZMAT3*, however this group did not show significant enrichment for any of the available clinical annotations. Finally, the heatmap did not show any association between IRS index and gene expression changes (Figure 4B).

### Identification of Biomarkers of Radiosensitivity

Univariate and multivariate statistical analyses were performed looking for association between (i) clinical variables of radiation toxicity and IRS index; (ii) clinical variables of radiation toxicity and expression level of DDR genes; (iii) IRS index and expression level of DDR genes.

**Figure 5:**
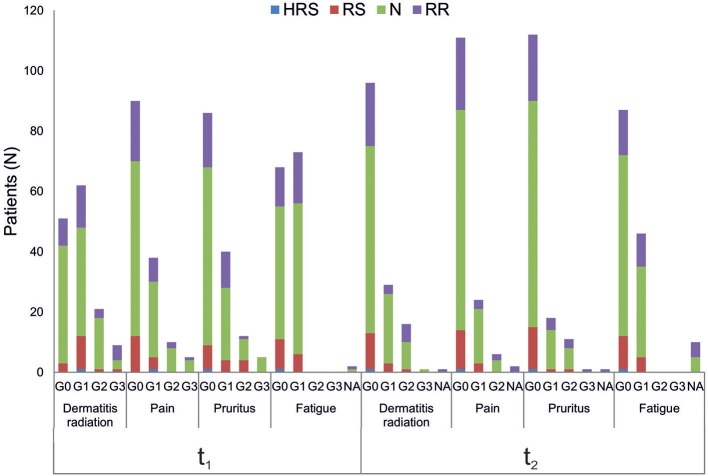
Association analysis between clinical variables of radiation toxicity and IRS index. Number of patients in the four IRS classes (HRS, RS, N, RR) per grades of radiation-induced adverse effects (G0, G1, G2, G3), at the completion of RT treatment (t_1_) and 1 month later (t_2_). NA, data Not Available.

Relationships between clinical variables of radiation toxicity and IRS classes are shown in Figure 5. At the completion of RT treatment (t_1_), patients experiencing adverse effects were distributed within the four IRS classes (HRS, RS, N, and RR), without any significant relationship and without differences between tumor types. Instead, for fatigue the IRS mean values significantly differed between patients with and without such adverse effect. Specifically, the estimated IRS mean values were, respectively, 40.44 and 36.17, with a decrease of 4.27 in patients with G1 fatigue at t_1_ (*p* = 0.015, *t-*test). The significance was confirmed by multivariate linear regression model (adjusted for age and disease type) (Table 2). Neither the multivariate nor the univariate analyses showed significant association between IRS index and clinical variables of radiation toxicity at t_2_.

**Table 2:** Multivariate linear regression model with IRS values and all clinical variables as covariates in 143 patients.

**Coefficients**	**Estimate**	**Std. error**	***t*-value**	**Pr(>|t|)**	
(Intercept)	39.123	5.804	6.740	4.86e-10	^***^
Tumor type—HNSCC vs. breast cancer	−1.023	2.967	−0.345	0.730	
Age in years	0.005	0.087	0.060	0.952	
Dermatitis radiation—G1 vs. G0	3.761	2.216	1.697	0.092	□
Dermatitis radiation—G2 vs. G0	−0.072	3.328	−0.022	0.982	
Dermatitis radiation—G3 vs. G0	−4.401	4.623	−0.952	0.342	
Pain—G1 vs. G0	−0.317	2.177	−0.146	0.884	
Pain—G2 vs. G0	−2.143	3.836	−0.559	0.577	
Pain—G3 vs. G0	1.216	6.050	0.201	0.8410	
Pruritus—G1 vs. G0	−1.764	2.324	−0.759	0.449	
Pruritus—G2 vs. G0	2.994	3.558	0.841	0.401	
Pruritus—G3 vs. G0	6.988	5.370	1.301	0.195	
Fatigue–G1 vs. G0	−4.265	1.960	−2.176	0.03	^*^

*IRS is considered as continuous value and clinical variables are those defined at the completion of RT treatment (t1). p-value of the model is 0.2217*.

*** p-value < 0.001,

* *p-value < 0.05*,

□ *p-value < 0.1*.

Significant and moderately significant associations between gene expression and clinical radiation toxicity are shown in Table 3. Dermatitis radiation at t_1_ was associated with a 1.88-fold change of *FDXR* expression in patients experiencing G3 toxicity vs. a 1.44-fold change in G2 patients. The presence of pain at t_1_ was associated with a decrease of *SESN1* expression (0.92- vs. 1.36-fold change in the comparison presence-absence, and 0.97- vs. 1.36-fold change when comparing more specifically G1 vs. G0). Symptoms of pruritus resulted associated at t_1_ with a 0.79-fold change of *XPC* and with a 1.01-fold change of *ZMAT3*; at t_2_ pruritus was associated with a 0.87-fold change of *ATM*, and a lower *BCL2/BAX* ratio (respectively, 0.76- vs. 1.15-fold change). G1 fatigue resulted associated with a 1.19-fold change of *CDKN1A* (p21) at the second clinical evaluation.

**Table 3:** DDR genes associated with clinical variables of radiation toxicity.

**Clinical variable**	**Gene**	**Gene expression value^a^**	**Toxicity grade**	**Adjusted *p-value***	**RT timing**
Dermatitis radiation	*FDXR*	1.44	G2	0.096^c^	t_1_
		1.88	G3		
Pain	*SESN1*	1.36	G0	0.043^b^	t_1_
		0.97	G1		
	*SESN1*	1.36	Absent	0.020^b^	t_1_
		0.92	Present		
Pruritus	*XPC*	1.49	G1	0.102^c^	t_1_
		0.79	G2		
	*ZMAT3*	1.43	G0	0.046^b^	t_1_
		1.01	G1		
	*ATM*	1.29	Absent	0.021^b^	t_2_
		0.87	Present		
	*BCL2/BAX*	1.15	Absent	0.011^b^	t_2_
		0.76	Present		
Fatigue	*CDKN1A*	1.42	G0	0.049^b^	t_2_
		1.19	G1		

a *Gene expression values are reported in irradiated relative to non-irradiated blood samples from RT patients and expressed in fold-change*.

b t-test;

c *Wilcoxon test. Bonferroni adjusted p-value is significant when <0.05, moderately significant when <0.10*.

By univariate analyses (Table 4) we found a moderate significant association between the RS class and *BBC3* and *FDXR* expression (adjusted *p* = 0.069) and between RR class and *GADD45A* expression (adjusted *p* = 0.096). The *BCL2/BAX* ratio was also associated with the RS class (adjusted *p* = 0.017).

**Table 4:** DDR genes associated with IRS classes in RT patients.

**Gene**	**Gene expression value^a^**	**IRS class**	**Adjusted *p-value***
*BBC3*	1.28	N	0.069^b^
	1.90	RS	
*FDXR*	1.53	N	0.069^b^
	1.84	RS	
*GADD45A*	1.19	N	0.096^c^
	1.73	RR	
*BCL2/BAX*	1.15	N	0.017^b^
	0.73	RS	

a *Gene expression values are reported in irradiated relative to non-irradiated blood samples from RT patients and expressed in fold-change*.

b t-test;

c *Wilcoxon test. Bonferroni adjusted p-value is significant when <0.05, moderately significant when <0.10*.

## Discussion

Despite the advancements in understanding and preventing RT effects on normal tissue, injuries deriving from radiation therapy cannot be avoided (33–35). Inter-individual differences in radiosensitivity are due to different endogenous and exogenous factors (e.g., DNA repair capacity, age, diet, and life-style) as well as to the experimental endpoint (clinical radiation toxicity, chromosome aberrations, etc.) (15, 27, 36, 37). Assessing the intrinsic component of radiosensitivity before RT could predict toxicity risk and improve the QOL (27, 28). To this purpose, from a cohort of oncological patients we collected data concerning radiation toxicity in normal tissues, *in vitro* G_2_-chromosomal radiosensitivity and *in vivo* expression level of 15 selected radioresponsive genes of DDR pathway, to find possible associations between genetic features and clinical radiosensitivity (Figure 1). By univariate and multivariate statistical models we have looked at significant associations between clinical and molecular data, controlling for potential confounders and for the multiplicity of the tests. Remarkably, no statistical differences have found between tumor types, allowing us to discuss our data as a whole.

To the best of our knowledge, this is the first study assessing the relationship between the three experimental endpoints in a cohort of RT-treated oncological patients. It is noteworthy that all patients have been enrolled in the same Radiotherapy Unit (IOV-IRCSS). Specifically, in this explorative study we considered breast and head and neck cancer patients as representative of patients experiencing radiation toxicity. Indeed, symptoms of grade 2 acute skin toxicity are observed in 15–24% of breast cancer patients at the completion of RT treatment (35, 38) whereas dermatitis radiation continues to be one of the most common side effects of RT in head and neck cancers (33, 39). In the present study, we considered dermatitis radiation, pain, pruritus and fatigue that are adverse effects commonly manifested in BC and HNSCC patients after RT, while tumor-specific adverse effects were excluded. Overall, HNSCC patients manifested higher degrees of dermatitis radiation and pain both at t_1_ and t_2_, whereas pruritus was more pronounced in BC patients at both t_1_ and t_2_ (Figure 2). For patients experiencing the highest level of dermatitis radiation we verified *ex post* the lack of relation with the phototype (Fitzpatrick scale). Fatigue induced by RT is a common symptom experienced by patients that deeply affects their QOL (40). In our cohort, all patients manifesting fatigue were evaluated as G1 grade, with overlapping proportions irrespective of cancer type: at t_1_ 50.8% (BC) and 57.8% (HNSCC), and at t_2_ 33.0 and 44.5%, respectively (Figure 2).

Previous studies showed that clinical radiation toxicity is related to G_2_-chromosomal radiosensitivity of *in vitro* irradiated lymphocytes (13, 19, 41). Here, we followed the standardized G_2_-assay developed by Pantelias and Terzoudi (15) in which the G_2_-checkpoint efficiency is abrogated by caffeine (inhibitor of ATM kinase) to maximize the radio-induced chromosomal damage, i.e., simulating the condition of high radiosensitivity of AT (Ataxia Telangiectasia) patients. This leads to accurate estimations of the individual radiosensitivity (the IRS index) by calculating the percentage ratio between the yields of radio-induced chromatid breaks in presence or absence of the functional G_2_-checkpoint (15, 29). IRS values obtained in the present study were distributed in strict agreement with previously published data (15, 29), confirming the reproducibility of the standardized G_2_-assay for assessing individual radiosensitivity *in vitro*. Based on multivariate analyses, fatigue emerged as the only adverse effect strictly associated with IRS index. Interestingly, in patients displaying G1 vs. G0 fatigue but having same values of other predictors, the average IRS index differed for a value of 4.27. No other clinical reactions were found associated with IRS values in this statistical analysis (Table 2).

Clinical radiosensitivity can be associated with individual factors, such as abnormal transcriptional responses to DNA damage and with defects in DNA repair (42–44). In this regard, previous studies of gene expression profiling, carried out in patient-derived PBLs irradiated *in vitro*, succeeded to some extent in discriminating groups of patients with and without severe late radiotherapy toxicity (23). An association was also observed between early skin reaction and the transcriptional response of lymphoblastoid cells derived from patients with acute radiation toxicity (4). The candidate genes here analyzed, belonging to the DNA Damage Response (DDR) pathway, were chosen on the basis of our previous data showing significant changes in their expression level in human PBLs at 24 h after irradiation with 2 Gy of γ-rays (30, 31). Moreover, *GADD45A, CDKN1A, DDB2* and *XPC*, together with *FDXR* gene are well-known radio-responsive genes (4, 26, 30, 31, 45, 46). Gene expression analyses have been carried out in 57 patients randomly selected within the cohort of 143 patients, as representative of the four IRS classes (HRS, RS, N, and RR). This sample size is adequate to identify significant differences of gene expression among the four IRS classes (statistical power 0.83), either by ANOVA or Wilcoxon test. The expression of DDR genes was on the whole significantly induced at 24 h after the first fractionated dose (Figure 4A), in accordance with their radioresponsiveness. Notably, *FDXR* expression showed a 1.6-fold increase, that is very similar to the ~1.7-fold increase reported at the same time point in four breast cancer and 8 HNSCC patients (26). In humans, *FDXR* expression is upregulated in a dose-dependent manner after irradiation, both *ex vivo* and *in vivo*, indicating that *FDXR* is a good biomarker for radiation exposure and for estimating *in vivo* dose (46–48). Moreover, *FDXR* belongs to a genetic signature for the early prediction of hematological acute radiation syndrome (47, 49). Unlike those authors who have found no association between *FDXR* expression and the hematological acute radiation syndrome in subjects undergoing RT, our work associates *FDXR* expression level with dermatitis radiation at the completion of RT treatment (G2 vs. G3 grades, adjusted *p* = 0.096) (Table 3). While the expression level of *FDXR* gene increased in patients experiencing a high grade of dermatitis radiation, those of *SESN1, ATM, XPC, ZMAT3, CDKN1A* genes decreased when radiation toxicity was manifested (Table 3). Of course, these findings may be explained by a more complex radiation response than that determined by the DDR genes here analyzed. Indeed, additional genes belonging to different pathways are expected to participate in the whole cellular response to radiation. Emerging evidence suggests that the response to radiation is differently regulated in normal vs. cancer cells/tissues, and even within organism, where maintaining the overall homeostasis is a priority (50, 51). Interestingly, the tight interplay between DDR and immune response seems a key feature shared by systems that differ for higher levels of complexity (51).

By integrating the data of chromosomal radiosensitivity and gene expression we found that IRS classes were associated with the expression level of three DDR genes. In particular, the increased expression of *BBC3* and *FDXR* genes, involved in the apoptotic pathway, is associated with the RS class, whereas the increased expression of *GADD45A*, regulating cell growth and apoptosis, is associated with the RR class (Table 4). A role of apoptotic pathway in normal tissue radiation toxicity has been previously reported, indeed the T-lymphocyte apoptosis assay significantly predicted differences in late radiation toxicity (52). Also the balance between pro-apoptotic and anti-apoptotic members of the BCL-2 family has a clinical significance on chemotherapy sensitivity and survival (53, 54). In our cohort, the *BCL2/BAX* ratio resulted associated both with the presence of pruritus 1 month after the completion of RT treatment (Table 3), and with the class of patients classified as radiosensitive by means of IRS index (Table 4). This common function is reinforcing the predictive value of these genes, although further analyses are necessary to support that they are reliable biomarkers of radiation toxicity.

Given the exploratory and pilot nature of our work, we privileged a cohort of RT patients, enrolled, treated and clinically evaluated in the same clinical Institute, irrespective of the tumor site. However, despite our multivariate analysis shows no statistical differences between BC and HNSCC patients (Table 2; Figure 4) future studies would be desirable to validate our results in a new cohort, taking into account additional clinical variables such as breast size, body mass index, alcohol consumption, hypertension, smoking habit, which might be associated to acute skin toxicity (55–57). Moreover, dosimetric data, radiation treatment volumes and doses to specific organs at risk would be important information to be included in future studies. Clearly, since HNSCC patients are less frequent than BC patients and often must undergo chemotherapy either before or concomitant to RT, multicentric studies would be recommended to reach a large sample size for both diseases.

## Conclusion

The possibility to identify patients that are sensitive to radiation and at risk of suffering adverse effects would help clinicians in tailoring the best RT protocol and improve patient's QOL. In this prospective cohort study, we found that symptoms of dermatitis radiation, pain, pruritus and fatigue were associated with the expression level of some genes of the DNA-damage response pathway (*FDXR, SESN1, XPC, ZMAT3, ATM, BCL2/BAX, and CDKN1A*). We also found that fatigue was significantly associated with IRS values; moreover, IRS classes resulted associated with the expression level of *BBC3, FDXR, GADD45A*, and *BCL2/BAX* genes.

Of course, radiation-induced side effects comprehend a complex cellular and tissue response that cannot be limited to the expression level of the DDR genes considered in this study, but it is rather regulated by a wide network of gene-interactions. The development of a reproducible and powerful assay to predict individual normal tissue radiosensitivity has been referred to as the “holy grail” of radiotherapy. Although several *in vitro* assays have been tested to identify reliable biomarkers able to predict normal tissue radiosensitivity, results obtained up to now are not informative enough. In this regard, our multidisciplinary approach can contribute to delineate the genetic features of patients manifesting different grades of radiation-induced toxicity.

## Data Availability Statement

The datasets analyzed during the current study are available from the corresponding authors on reasonable request.

## Ethics Statement

The studies involving human participants were reviewed and approved by IOV-IRCCS Ethic Committee. The patients/participants provided their written informed consent to participate in this study.

## Author Contributions

LC, DZ, AR, and MM conceived and designed the study. EF, EG, FB, BE, and MR contributed in enrollment of patients, radiotherapy treatment, and clinical follow-up. EP and CP carried out the experiments of gene expression and participated in data evaluation. LB carried out the experiments of G2-chromosomal assay. EC and CR performed the statistical analyses and participated in data evaluation. MM and AR wrote the final manuscript. The definitive supervision of the paper was done by LC. All authors contributed to the initial draft, read, and approved the final manuscript.

### Conflict of Interest

The authors declare that the research was conducted in the absence of any commercial or financial relationships that could be construed as a potential conflict of interest.

## Abbreviations

CTCAE: Common Terminology Criteria for Adverse Events
DDR: DNA-Damage Response
DSBs: Double-Strand Breaks
HNSCC: Head and Neck Squamous Cell Carcinoma
HRS: Highly Radiosensitive
IR: Ionizing Radiation
IRS: Individual Radiosensitivity
N: Normal
PBLs: Peripheral Blood Lymphocytes
QOL: Quality of Life
qRT-PCR: quantitative Real-Time PCR
RR: Radioresistant
RS: Radiosensitive
RT: Radiotherapy.
