# Supplementary material for: Individual Radiosensitivity in Oncological Patients: Linking Adverse Normal Tissue Reactions and Genetic Features
Source: Front Oncol. 2019 Oct 1;9:987. doi: 10.3389/fonc.2019.00987 (PMC6779824; doi:10.3389/fonc.2019.00987)
Supplement: Supplementary file 1 [file Data_Sheet_1.PDF]

Supplementary Table 1. Sequence of primers used for gene expression analyses by qRT-PCR.

**Primer name**	**Primer sequence (5'-->3')**	**ref. mRNA**
**ATM_FOR**	ACTGGCCAGAACTTTCAAGAAC	NM_000051.3
**ATM_REV**	TGCCCAGAATACTTGTGCTTC	
**BAX_FOR**	GAACCATCATGGGCTGGA	NM_001291428.1
**BAX_REV**	CGTCCCAAAGTAGGAGAGGA	
**BBC3_FOR_4**	GACCTCAACGCACAGTACGA	NM_014417.4
**BBC3_REV_4**	GAGATTGTACAGGACCCTCCA	
**BCL2_FOR**	GTGGATGACTGAGTACCTGAACC	NM_000633.2
**BCL2_REV_2**	AGAGACAGCCAGGAGAAATCAAA	
**CCNG1_FOR**	TACTGCCTCAAACTGAATCCCAT	NM_004060.3
**CCNG1_REV**	TACCTTAGTGTGGGAAAGCAAGT	
**cMYC_FOR**	CACCAGCAGCGACTCTGA	NM_002467.4
**cMYC_REV**	GATCCAGACTCTGACCTTTTGC	
**DDB2_FOR**	TGGCATCAGTTCGCTTAATG	NM_000107.2
**DDB2_REV**	CTGGCCCACACCTTCTTTAG	
**FDXR-3'UTR_FOR**	CGCAAGGTTTTAGCTTTCAGCA	NM_024417.4
**FDXR-3'UTR_REV**	GCAGTAGAGAGATGGGTAAGGGG	
**GADD45A_FOR**	GCTGGTGACGAATCCACAT	NM_001924.3
**GADD45A_REV**	AGATGCCATCACCGTTCAG	
**GAPDH_FOR**	TCCTCTGACTTCAACAGCGA	NM_002046.5
**GAPDH_REV**	GGGTCTTACTCCTTGGAGGC	
**MDM2_FOR**	AGTGAAGAAGGACAAGAACTCTC	NM_001145340.2
**MDM2_REV**	TTTTCCTCAACACATGACTCTCT	
**p21_FOR (CDKN1A)**	TTAGCAGCGGAACAAGGAGT	NM_000389.4
**p21_REV**	CAACTACTCCCAGCCCCATA	
**PCNA_FOR**	CTCAAGAAGGTGTTGGAGGC	NM_002592.2
**PCNA_REV**	GTAGGTGTCGAAGCCCTCAG	
**SESN1_FOR**	CTTAGACGGGCAATTTGGAACTA	NM_014454.2
**SESN1_REV**	CATGAACCTTCTCAGAGTGCTTG	
**XPC_FOR**	GCCTCAAAACCGAGAAGATG	NM_004628.4
**XPC_REV**	CAGCTTCTCAAATGGGAACA	
**ZMAT3_FOR**	TCAGCAGGTCCTTACTTCAATCC	NM_022470.3
**ZMAT3_REV**	GCTCCAACATTACACATTGAGCA	
