# Supplementary material for: Individual Radiosensitivity in Oncological Patients: Linking Adverse Normal Tissue Reactions and Genetic Features
Source: Front Oncol. 2019 Oct 1;9:987. doi: 10.3389/fonc.2019.00987 (PMC6779824; doi:10.3389/fonc.2019.00987)
Supplement: Supplementary file 2 [file Data_Sheet_2.PDF]

Supplementary Table 2. Adverse effects, recorded according the CTCAE criteria version 4.03, in the cohort of 143 patients enrolled in this study. NA = data Not Available.

	**Adverse effect**	**CTCAE Grade**	**BC patients (N)**	**HNSCC patients (N)**
**t_1**	Dermatitis radiation	G0	42	9
		G1	58	4
		G2	17	4
		G3	7	2
	Pain	G0	80	10
		G1	36	2
		G2	7	3
		G3	1	4
	Pruritus	G0	69	17
		G1	39	1
		G2	12	0
		G3	4	1
	Fatigue	G0	60	8
		G1	62	11
		G2	0	0
		G3	0	0
		NA	2	0
**t_2**	Dermatitis radiation	G0	90	6
		G1	23	6
		G2	10	6
		G3	1	0
		NA	0	1
	Pain	G0	101	10
		G1	20	4
		G2	3	3
		G3	0	0
		NA	0	2
	Pruritus	G0	98	14
		G1	15	3
		G2	11	0
		G3	0	1
		NA	0	1
	Fatigue	G0	77	10
		G1	38	8
		G2	0	0
		G3	0	0
		NA	9	1
