# Supplementary material for: Individual Radiosensitivity in Oncological Patients: Linking Adverse Normal Tissue Reactions and Genetic Features
Source: Front Oncol. 2019 Oct 1;9:987. doi: 10.3389/fonc.2019.00987 (PMC6779824; doi:10.3389/fonc.2019.00987)
Supplement: Supplementary file 3 [file Data_Sheet_3.PDF]

Supplementary Table 3. Clinical data and IRS classification of the 57 patients included in the gene expression analyses.

			Patients		
			**BC**	**HNSCC**	**Total**
			50	7	57
	**IRS classes**	RR	8	1	9
		N	29	6	35
		RS	11	0	11
		HRS	1	0	1
		NA	1	0	1
**t_1**	**Dermatitis radiation**	G0	25	6	31
		G1	14	0	14
		G2	7	1	8
		G3	4	0	4
	**Pain**	G0	40	5	45
		G1	9	1	10
		G2	1	1	2
		G3	0	0	0
	**Pruritus**	G0	29	6	35
		G1	15	0	15
		G2	3	0	3
		G3	3	1	4
	**Fatigue**	G0	33	4	37
		G1	17	3	20
		G2	0	0	0
		G3	0	0	0
**t_2**	**Dermatitis radiation**	G0	36	1	37
		G1	8	3	11
		G2	6	3	9
		G3	0	0	0
	**Pain**	G0	45	3	48
		G1	2	2	5
		G2	2	2	4
		G3	0	0	0
	**Pruritus**	G0	40	4	44
		G1	7	2	9
		G2	3	0	3
		G3	0	1	1
	**Fatigue**	G0	32	4	36
		G1	18	3	21
		G2	0	0	0
		G3	0	0	0
